# Evaluating the effects of giraffe skin disease and wire snare wounds on the gaits of free-ranging Nubian giraffe

**DOI:** 10.1038/s41598-023-28677-y

**Published:** 2023-02-03

**Authors:** L. M. Bernstein-Kurtycz, N. T. Dunham, J. Evenhuis, M. B. Brown, A. B. Muneza, J. Fennessy, P. M. Dennis, K. E. Lukas

**Affiliations:** 1 Division of Conservation and Science, Cleveland Metroparks Zoo, 4200 Wildlife Way, Cleveland, OH 44109 USA; 2 Department of Biology, Case Western Reserve University, Cleveland, OH USA; 3 Little Rock Zoo, Little Rock, AR USA; 4 College of Veterinary Medicine and Biomedical Sciences, Colorado State University, Fort Collins, CO USA; 5 Giraffe Conservation Foundation, P.O. Box 86099, Eros, Namibia; 6 Smithsonian National Zoo and Conservation Biology Institute, Front Royal, VA 22630 USA; 7 Department of Biological Sciences Program in Ecology, Evolution, Ecosystems, and Society, Dartmouth College, Hanover, NH USA; 8 Department of Veterinary Preventive Medicine, The Ohio State University, Columbus, OH USA

**Keywords:** Ecology, Zoology

## Abstract

Giraffe skin disease (GSD), a condition that results in superficial lesions in certain giraffe (*Giraffa* spp.) populations, has emerged as a potential conservation threat. Preliminary findings suggested that individuals with GSD lesions move with greater difficulty which may in turn reduce their foraging efficiency or make them more vulnerable to predation. A current known threat to some giraffe populations is their mortality associated with entrapment in wire snares, and the morbidity and potential locomotor deficiencies associated with wounds acquired from snares. The goal of our study was to quantify the locomotor kinematics of free-ranging Nubian giraffe (*G. camelopardalis camelopardalis*) in Murchison Falls National Park (MFNP), Uganda, and compare spatiotemporal limb and neck angle kinematics of healthy giraffe to those of giraffe with GSD lesions, snare wounds, and both GSD lesions and snare wounds. The presence of GSD lesions did not significantly affect spatiotemporal limb kinematic parameters. This finding is potentially because lesions were located primarily on the necks of Nubian giraffe in MFNP. The kinematic parameters of individuals with snare wounds differed from those of healthy individuals, resulting in significantly shorter stride lengths, reduced speed, lower limb phase values, and increased gait asymmetry. Neck angle kinematic parameters did not differ among giraffe categories, which suggests that GSD neck lesions do not impair normal neck movements and range of motion during walking. Overall, MFNP giraffe locomotor patterns are largely conservative between healthy individuals and those with GSD, while individuals with snare wounds showed more discernible kinematic adjustments consistent with unilateral limb injuries. Additional studies are recommended to assess spatiotemporal limb kinematics of giraffe at sites where lesions are found predominantly on the limbs to better assess the potential significance of GSD on their locomotion.

## Introduction

Once widely distributed across the continent of Africa, giraffe (*Giraffa* spp.) have declined in both distribution and abundance over the last century due to habitat loss and fragmentation, civil unrest, poaching (i.e., illegal hunting), and ecological change^1–4^. Giraffe populations experienced an overall ~ 30% decline in the last three and a half decades, and today there are an estimated 117,000 giraffe in the wild^3^. In 2016, the International Union for Conservation of Nature (IUCN) up-listed giraffe as a single species (i.e., *Giraffa camelopardalis*) from “Least Concern” to “Vulnerable” on the Red List, emphasizing the population declines and severity of threats facing them. The taxonomic classification of giraffe is a topic of debate^5–12^; however, here we utilize the classification that recognizes four taxonomically distinct species: Masai giraffe (*G. tippelskirchi*), reticulated giraffe (*G. reticulata*), northern giraffe (*G. camelopardalis*), and southern giraffe (*G. giraffa*)^7,8,12^. The conservation status of three species (*G. tippelskirchi*, *G. camelopardalis*, and *G. reticulata)* are of great concern, with their numbers all declining by > 50% and absent from much of their estimated historic geographical ranges^3,13^. Giraffe play a key role in shaping the ecology of savannah and woodland ecosystems, and their loss across the continent could have far-reaching long-term ecological consequences^14,15^.

**Figure 1:**
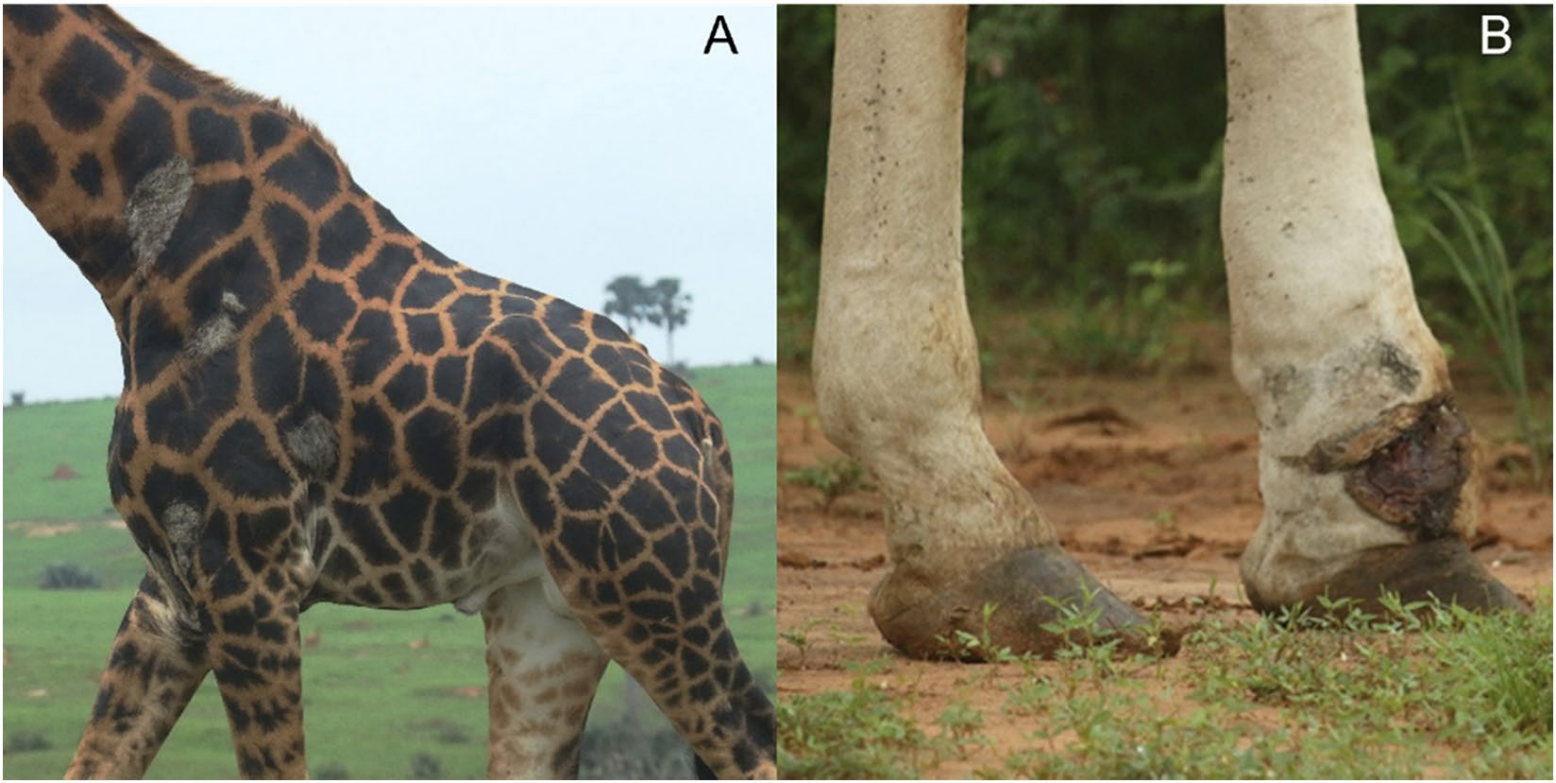
Images of giraffe skin disease (**A**) and wire snare wounds (**B**) in Nubian giraffe *Giraffa camelopardalis camelopardalis* from Murchison Falls National Park, Uganda. Photo credit: Michael B. Brown.

In the mid-1990’s, giraffe skin disease (GSD) emerged as a new potential threat to giraffe conservation. GSD was first described in a population of Nubian giraffe (*G. c. camelopardalis*) in Uganda^16^. Since then, GSD has been detected in at least 13 parks and reserves across seven countries in Africa, including South Africa, Botswana, Namibia, Zimbabwe, Kenya, Tanzania, and Uganda^17^. Prevalence of GSD has been reported to be as high as 86% in Masai giraffe inhabiting Ruaha National Park (NP), Tanzania^18–20^, and at a given time, as many as 51% of giraffe in Murchison Falls NP (MFNP) in Uganda may be afflicted with visible signs of GSD (M. Brown, pers. comm., May 2021). Furthermore, skin conditions resembling GSD have been reported in 11 zoos in six countries, including Belgium, England, France, Italy, the Netherlands, and the United States^17^. We now know that GSD manifests as different skin conditions; however, all GSDs generally manifest as large, crusted, scab-like wounds, sometimes with accompanying cracks in the skin, appearing mainly on the limbs, chest, and neck, though location of lesions differs by geographical location^17,18,21–23^ (Fig. 1A).

Several etiological agents have been suggested in the pathogenesis of different GSDs, including bacterial, fungal, and protozoal organisms, although current research suggests a nematode origin, with possible accompanying fungal infection^18,23,24^. Further, the mode of transmission of GSDs remains unknown. In some populations, severe forms of infection could make afflicted individuals more prone to predation because it may affect locomotion. Researchers have noted that infected animals seem to move with difficulty or suffer from lameness^18^; however, no studies to date have systematically studied the effects of GSDs on giraffe locomotion. As MFNP giraffe travel an average of ~ 14 km per day (M. Brown, pers. comm., May 2021), a disease that affects locomotor mechanics and efficiency could have serious consequences for an individual’s ability to seek resources^25^ and evade predators^26,27^.

Another threat to some giraffe populations is morbidity associated with snare-related injuries and potential mortality caused by entrapment in wire snares (Fig. 1B). The increasing illegal harvesting of bushmeat is a significant cause of population declines for many wildlife species across sub-Saharan Africa, and the use of wire snares is a common technique^28^. The relatively indiscriminate nature of snares results in a considerable amount of unintended bycatch^29,30^, which includes giraffe^4,31^. In addition to the mortality associated with poaching, injuries associated with escaping from snares have been shown to affect locomotion and behavior in other taxa^32^ and have been linked to increased parasite load^33^. In giraffe, modified locomotion and behavior could potentially increase predation risk and decrease foraging efficiency. In this way, evaluating the effects of snare wounds on giraffe locomotion could lead to a deeper mechanistic understanding of snare-related mortality and morbidity.

A handful of studies have examined the locomotion and gait of giraffes^34–39^. These important studies provide the groundwork for our investigation. Recently, Basu et al.^39^ quantified spatiotemporal limb kinematics, kinetics (i.e., ground reaction forces), and neck angle kinematics of walking gaits in three adult zoo-housed reticulated giraffe. They found that giraffe walking gaits can be classified as lateral sequence, lateral couplets (LSLC, i.e., a gait in which the touchdown of a hindlimb is followed shortly thereafter by a touchdown of the ipsilateral forelimb). This gait type helps to avoid limb interference in which limbs contact one another during the stride but differs from a true “pace” gait where the touchdowns and liftoffs of ipsilateral limbs is simultaneous or approximately simultaneous^40^. The movement of the neck is functionally linked to the giraffe walking gait, with horizontal acceleration of the neck out of phase with the horizontal acceleration of the trunk^39^.

The goal of our study was to quantify the spatiotemporal limb kinematics and neck angle kinematics in free-ranging Nubian giraffe in MFNP. In doing so, we compared kinematic parameters of healthy free-ranging giraffe (i.e., no visible GSD nor visible snare wounds) to those of free-ranging giraffe with GSD, snare wounds, and both GSD and snare wounds. Based on field observations that some giraffe with GSD were found to walk with greater difficulty^18^, we predicted that the spatiotemporal limb kinematics and neck angle kinematics of individuals with GSD would differ from those of healthy individuals. We also examined the mechanisms through which snare wounds impacted giraffe locomotion and predicted that, compared to healthy giraffe, individuals with snare wounds would exhibit slower walking speeds and kinematic adjustments consistent with reduced loading on the injured limb^41^. To our knowledge, our study is the first to quantify walking gait kinematics in free-ranging giraffe and the first to test whether and how GSD and snare wounds affect giraffe locomotion. Given the prevalence of GSD and intense wire snaring pressure on giraffe in MFNP, gaining a better understanding of how GSD and snare wounds impact locomotor capabilities of afflicted giraffe will provide important context for assessing broader health and fitness.

## Methods

### Subjects and video recordings

### Preliminary data collection at Cleveland Metroparks Zoo

We first developed and piloted our methods in a zoo setting at Cleveland Metroparks Zoo, Ohio, USA, before applying them to videos of free-ranging Nubian giraffe from MFNP. We analyzed the gait of four Masai giraffe at Cleveland Metroparks Zoo including: one adult male (aged eight) and three adult females (aged seven, nine, and ten). The subjects were known to be in good health during the time of observation. We filmed giraffe at 30 frames per second walking over flat ground using a Casio EX-FC150 camera. Thirteen video clips containing 31 strides were suitable for spatiotemporal limb kinematic analyses (i.e., all footfalls visible), and seven strides were suitable for neck angle kinematic analyses (i.e., giraffe moving approximately perpendicular to the camera).

### Murchison Falls National Park (MFNP)

We collected data on 52 adult male giraffe in MFNP (2.1458° N, 31.8069° E) from July 2015–August 2016. In association with a long-term study designed to evaluate the demography and spatial ecology of male giraffe in MFNP, a series of fixed route individual based photographic surveys were conducted over the entire extent of the park^42^. We identified each individual using their unique pelage patterns^43^. Videos were recorded opportunistically during ongoing conservation science research, which included assessing the etiology of GSD locally^23^. All videos were recorded at ~ 75–100 m from the focal animal. Individuals were recorded on flat ground to control for gait adjustments driven by changes in terrain and inclination/declination. Each giraffe was assigned to one of four condition categories: (1) healthy (i.e., no visible GSD lesions nor snare wounds), (2) GSD (presence of GSD lesions), (3) snare (presence of snare wound), or (4) GSD and snare (presence of both GSD lesion(s) and snare wound). We acknowledge that giraffe assigned to our healthy category may have underlying health issues other than GSD, and some historical snare wounds are not easily observable; however, we assumed these potential factors did not significantly impact the kinematic variables examined in our study. Additionally, we recognize that researchers have categorized the severity of GSD^18,22^; however, due to limitations with our sample size, we opted to report presence vs. absence of GSD as other studies have done^19,44^.

Giraffe locomotion at MFNP was filmed at 30 frames per second using either a Nikon CoolPix AW110 or a Canon 7D Mark II digital camera. We selected only videos with clear, unobstructed views of limb touchdowns (n = 32 videos). The videos included in the spatiotemporal gait analyses ranged from eight to 49 s and contained between one and eight strides per individual (n = 115 strides). We attempted to film perpendicular to the line of travel when possible; however, parallax is not an issue for timing and digitizing limb touchdown and liftoff events. Furthermore, our spatial points (i.e., stride length and shoulder height) were all digitized in the same video frame for a given video clip, eliminating potential distortion due to parallax issues. That is, all in-plane linear distance metrics would suffer a similar degree of distortion, allowing ratios of these distances to be unbiased^45^. Because angular measurements are susceptible to parallax issues, only strides in which giraffe were moving approximately perpendicular to the camera were used to quantify neck angle measurements (n = 71 strides) (Table 1).

**Table 1:** Number of strides and individuals for different condition categories in free-ranging Nubian giraffe *Giraffa camelopardalis camelopardalis* from Murchison Falls National Park, Uganda.

Condition	Number of strides (number of individuals)	
	Spatiotemporal limb kinematics	Neck angle kinematics
Healthy	34 (10)	22 (8)
GSD	31 (10)	25 (10)
Snare	18 (4)	9 (3)
GSD and snare	32 (9)	15 (8)
Total	115 (33)	71 (29)

### Digitizing methods

**Figure 2:**
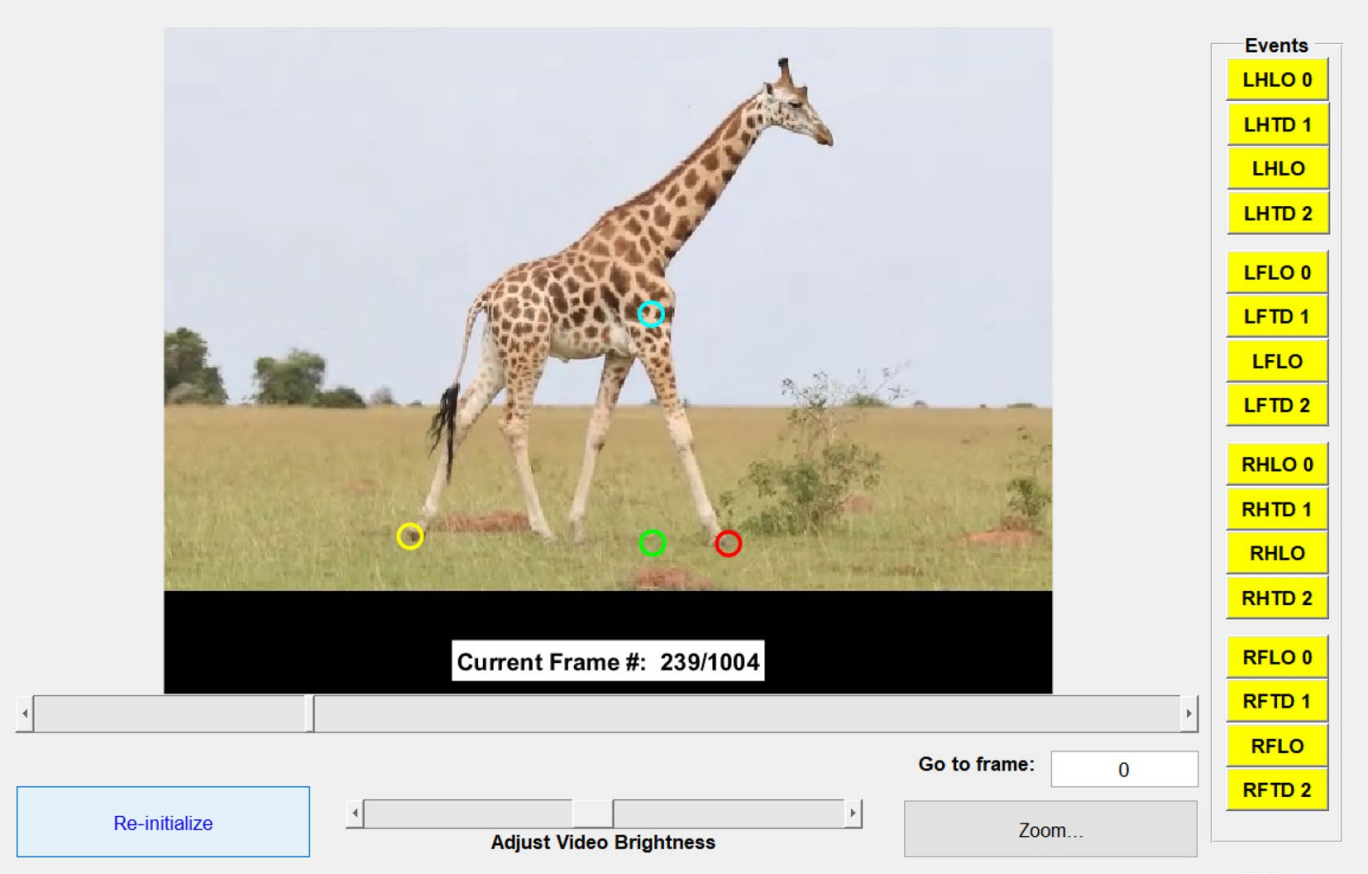
Screenshot of GaitKeeper graphical user interface used to digitize spatiotemporal limb kinematics. The timings of limb liftoff (LO) and touchdown (TD) events were recorded by selecting the corresponding video frame number for each event. We digitized two LO and TD events for each limb (i.e., LH = left hindlimb; LF = left forelimb; RH = right hindlimb; RF = right forelimb). These data were used to quantify stride duration, limb phase, mean number of supporting limbs, and duty factors. Stride length (i.e., distance between yellow and red points) and shoulder height (i.e., distance between blue and green points) were quantified in pixels and used to calculate relative stride length. Stride duration and relative stride length were used to calculate relative speed.

We used GaitKeeper, an open-source MATLAB package, to digitize limb liftoff and touchdown events, stride length, and shoulder height^45^ (Fig. 2; http://www.younglaboratory.org/GaitKeeper). We recorded neck angle measurements for individual videoframes using the angle tool in ImageJ^46^.

### Kinematic variables

#### Spatiotemporal limb kinematics

We quantified giraffe shoulder height (in pixels) by digitizing a point at roughly the height of the glenohumeral joint and another point at ground level directly below the shoulder. We quantified stride length (in pixels) by digitizing the initial touchdown of a reference limb (e.g., left hindlimb) and the subsequent reference limb touchdown. Relative stride length was calculated by dividing stride length in pixels by shoulder height in pixels. Relative (i.e., dimensionless) stride length was reported to account for differences in body size among giraffe. We predicted that giraffe with GSD and/or snare wounds would have shorter relative stride lengths than healthy individuals^41^.

Stride duration was recorded as the amount of time (in seconds) between the initial and subsequent touchdown of a reference limb. We used stride durations for each limb to generate mean stride duration. We predicted that giraffe with GSD and/or snare wounds would have greater mean stride durations than healthy individuals^41^.

We calculated relative speed by dividing relative stride length by mean stride duration, resulting in values with units of % of shoulder height per second. Relative speed was reported to control for potential speed differences due to differences in body size among giraffe. We predicted that giraffe with GSD and/or snare wounds would move at slower relative speeds compared to healthy individuals^41^.

We quantified footfall patterns according to limb phase which is defined as the proportion of stride duration separating hindlimb touchdown from ipsilateral forelimb touchdown during symmetrical gaits. Limb phase patterns are associated with stability during movement^47^ and vary among species due to differences in anatomy and habitats and can vary within a species or individuals depending on the speed of movement and the substrate or terrain in which the animal is moving. These limb phase values are often used to classify symmetrical gaits such that limb phase values between 0 and 25% are designated as LSLC gaits, values between 25 and 50% are lateral sequence, diagonal couplet (LSDC) gaits, values between 50 and 75% are diagonal sequence, diagonal couplet (DSDC) gaits, and values between 75 and 100% are diagonal sequence, lateral couplets (DSLC) gaits. While researchers utilize different thresholds for named gait types, limb phase values equal to or approximately equal to 0%, 25%, 50%, and 75% are classified as pace, lateral sequence singlefoot, trot, and diagonal sequence singlefoot gaits, respectively^40,47,48^. Our preliminary data revealed that Masai giraffe housed at Cleveland Metroparks Zoo exclusively used LSLC walking gaits—a pattern consistent with another recent study of zoo-housed reticulated giraffe^39^. We predicted that free-ranging Nubian giraffe would also use LSLC gaits and tested whether limb phase values for giraffe with GSD and/or snare wounds deviated from the limb phase values of healthy giraffe.

The mean number of supporting limbs (NSL) can theoretically vary between zero (i.e., aerial phase) and four (i.e., stationary animal) throughout different portions of a stride. We quantified the portion of stride duration in which individuals were supported by zero, one, two, three or four limbs, to generate mean NSL throughout the stride^49–51^. Controlling for speed, we predicted that giraffe with snare wounds would have greater mean NSL compared to healthy individuals as a strategy to reduce loading on the affected limb^41^.

We quantified duty factor (i.e., the amount of time a limb is in contact with the ground divided by stride duration) for each of the four limbs. We then calculated an asymmetry index for ipsilateral duty factors (ipsilateral DFAI) modified from Robinson et al.^52^ and Vanden Hole et al.^53^ such that: ((L_df_ − R_df_)/(0.5 (L_df_ + R_df_)) × 100, where L_df_ = mean of left forelimb and left hindlimb duty factors and R_df_ = mean of right forelimb and right hindlimb duty factors. Values can theoretically range from − 100 to 100% with 0% indicating perfect contralateral symmetry. More negative values indicate lower duty factors on the left limbs compared to the right limbs. More positive values indicate lower duty factors on the right limbs compared to the left limbs. For animals with unilateral snare injuries, we modified the equation such that the injured/affected limb would be considered first. That is, for animals with right limb snare wounds, we used ((R_df_ − L_df_)/(0.5 (R_df_ + L_df_)) × 100. Thus, more negative values indicate lower duty factor on the side of the injured limb and more positive values indicate greater duty factors on the side of the injured limb. Because giraffe are typically supported by ipsilateral limb couplets for long periods of stride duration^39^, we predicted that individuals with unilateral snare injuries would have more negative ipsilateral DFAI values due to reduced contact time on the side of the injured limb as part of a pattern to reduce loading on that limb^41,54^. In contrast, we predicted healthy individual would have ipsilateral DFAI values equal to or close to zero (i.e., consistent with contralateral symmetry).

#### Neck angle kinematics

**Figure 3:**
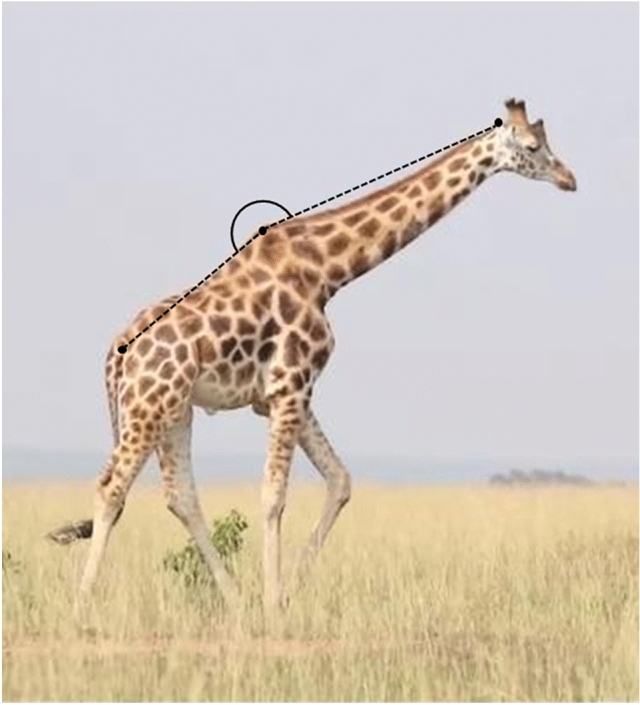
Adult male Nubian giraffe (*Giraffa camelopardalis camelopardalis*) from Murchison Falls National Park, Uganda. Black dots indicate the three points digitized to generate neck angle during peak dorsal extension and peak ventral flexion. This figure indicates peak ventral flexion of the neck which coincides with right forelimb midstance.

A giraffe’s neck oscillates twice during a typical walking stride with peak dorsal extension (i.e., neck more vertical) occurring during early stance phase of each forelimb and peak ventral flexion (i.e., neck more horizontal) occurring at roughly midstance of each forelimb^35,39^. Basu et al.^39^ found that the horizontal acceleration of the neck was largely out of phase with the acceleration with the trunk which likely allows giraffe to move more efficiently (i.e., similar to how a crouched jockey can horizontally oscillate their body out of phase with a race horse’s body thereby reducing the mass the horse has to accelerate and decelerate in the horizontal plane). This ultimately improves the horse’s locomotor performance^55^. Given the importance of the neck during giraffe locomotion, we quantified neck angle during peak dorsal extension and peak ventral flexion for each stride. Points located at the base of the tail, apex of the dorsal spinous processes of the thoracic vertebrae (i.e., “withers”), and apex of the occipital bone posterior to the ossicones were used to generate the neck angle. These landmarks were chosen because they were easily observable on the giraffe from our video sample (Fig. 3) and are similar to those used previously^35,39^. We digitized neck angle in each video frame using ImageJ^46^ and identified the minimum and maximum values as peak dorsal extension and peak ventral flexion, respectively. We then calculated neck range of motion (ROM) as the difference between peak ventral flexion and peak dorsal extension following Basu et al.^39^.

#### Statistical analyses

We used linear mixed models to examine the effect of giraffe condition on spatiotemporal limb kinematics variables, including relative stride length, mean stride duration, relative speed, limb phase, mean NSL, and ipsilateral DFAI. Similarly, we used linear mixed models to assess the effect of giraffe condition on neck angle kinematics, including peak dorsal extension, peak ventral flexion, and neck ROM. For all statistical models, we included GSD (presence vs. absence) and snare wound (presence vs. absence) as fixed factors. Individual giraffe were nested within video clip as a random factor (intercept) in each model to control for random variation between individuals, providing greater power to detect meaningful variation associated with the fixed factors of interest (i.e., presence vs. absence of GSD and snare wound). Satterthwaite approximations were used to adjust degrees of freedom in cases of heteroscedasticity for all models relating giraffe condition to spatiotemporal limb kinematics and neck angle kinematics. Analyses were conducted in R statistical software^56^, including add-on packages: lme4 and lmerTest^57^. We used the emmeans package^58^ to generate estimated marginal mean values for each dependent variable listed above. Post hoc pairwise comparisons of mixed models were conducted using the emmeans package, with multiple pairwise comparisons corrected using the false discovery rate method^59^. Relative speed was included as a covariate in the mean NSL and neck ROM models because speed has been shown to negatively correlate with mean NSL in other taxa^51^ and positively correlate with neck ROM in zoo-housed giraffe^39^.

## Results

### Zoo vs. field data

We found that the spatiotemporal limb kinematics and neck angle kinematics of the zoo-housed Masai giraffe were generally similar to those recorded in healthy free-ranging Nubian giraffe (Table 2). Given the two datasets were collected on different giraffe species occupying different environments, and the fact that the zoo sample contained both an adult male and three adult female giraffe and the free-ranging samples contained only adult male giraffe, we did not statistically compare the two datasets. Nonetheless, the similar results suggest that our methods developed at Cleveland Metroparks Zoo were transferrable to field conditions.

**Table 2:** Mean spatiotemporal limb kinematics and neck angle kinematics of four adult (n = 3 females and 1 male) Masai giraffe (*Giraffa tippelskirchi*) housed at Cleveland Metroparks Zoo (n = 31 strides for limb kinematics and n = 7 strides for neck angle kinematics) vs. those of healthy free-ranging Nubian giraffe (*Giraffa camelopardalis camelopardalis*) from Murchison Falls National Park, Uganda (n = 34 strides for limb kinematics and n = 22 strides for neck angle kinematics). Standard deviations are listed in parentheses.

Parameter	Zoo giraffe	Healthy MFNP giraffe
Spatiotemporal limb kinematics		
Relative stride length	1.13 (0.10)	1.26 (0.10)
Mean stride duration (s)	2.18 (0.10)	2.31 (0.13)
Relative speed	0.52 (0.06)	0.55 (0.06)
Limb phase (%)	14.77 (1.26)	13.10 (1.28)
Mean NSL	2.56 (0.05)	2.72 (0.05)
Ipsilateral DFAI (%)	2.59 (1.84)	− 1.22 (3.58)
Neck angle kinematics		
Peak dorsal extension (°)	180.1 (6.3)	181.0 (7.2)
Peak ventral flexion (°)	190.1 (5.1)	195.9 (8.0)
ROM (°)	10.0 (2.7)	14.9 (4.6)

### Effects of giraffe condition on spatiotemporal limb kinematics

**Figure 4:**
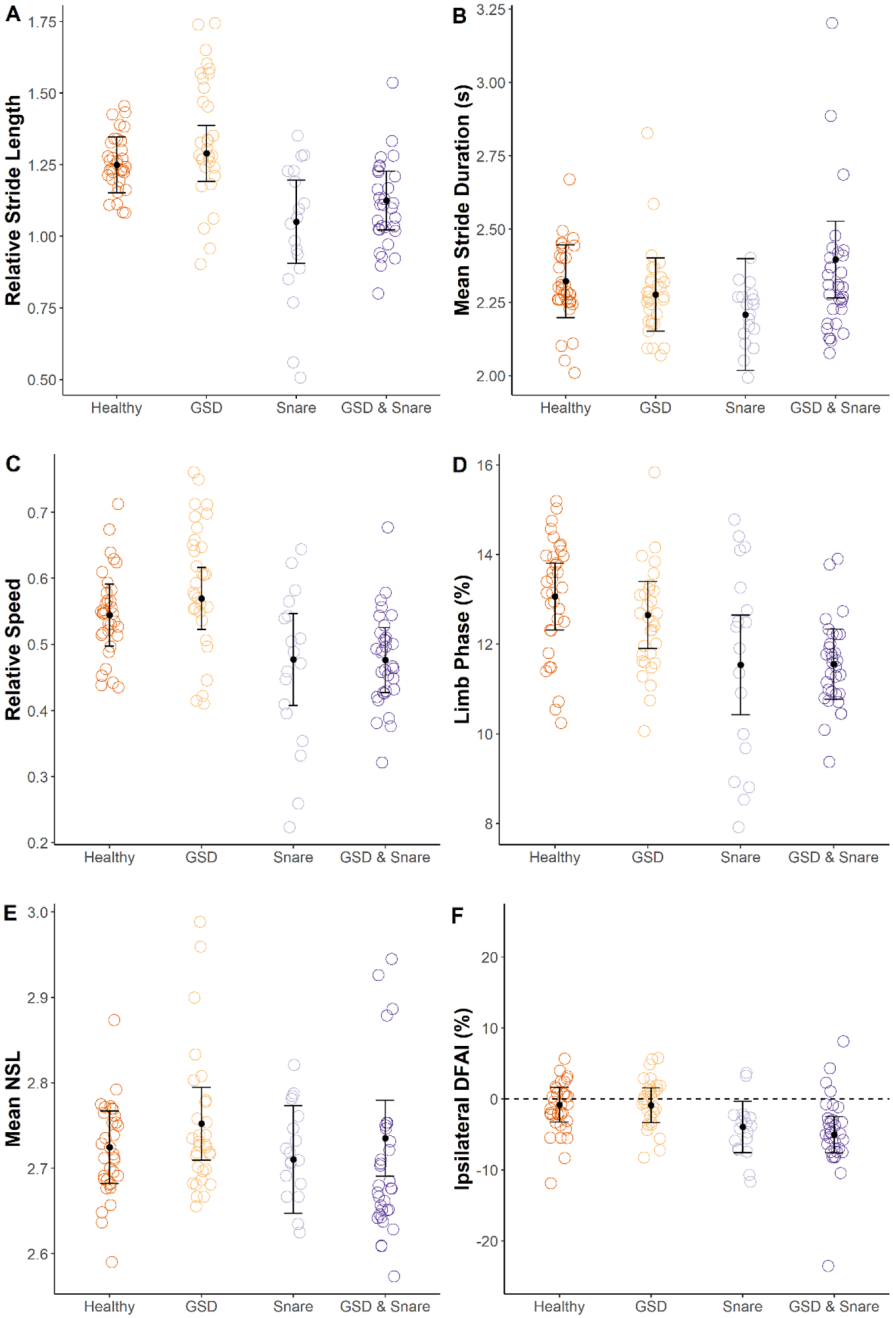
Variation in spatiotemporal limb kinematics among different conditions (i.e., healthy, GSD, snare wound, and both GSD and snare wound) in free-ranging Nubian giraffe *Giraffa camelopardalis camelopardalis* from Murchison Falls National Park, Uganda. Each point represents a stride. Black dots indicate estimated marginal means and black error bars indicate 95% confidence intervals from the linear mixed models. Black dotted line in panel F indicates contralateral symmetry.

We found that GSD lesions were primarily located on the necks of giraffe in our sample (Table 3). Snare wounds were observed more frequently on one of the hindlimbs (n = 9) compared to one of the forelimbs (n = 4) (Table 3). The presence of snare wounds affected multiple aspects of spatiotemporal limb kinematics while, contrary to our predictions, the presence of GSD did not significantly affect any of the spatiotemporal limb kinematic variables investigated (Table 4). When examining post hoc multiple pairwise comparisons of estimated marginal means, individuals with only snare wounds had significantly shorter relative stride lengths (p = 0.006), slower relative speeds (p = 0.009), lower limb phase values (p = 0.01), and more negative ipsilateral DFAI (p = 0.02) compared to healthy giraffe. Similarly, individuals with both GSD and snare wounds had lower limb phase values (p = 0.04), and more negative ipsilateral DFAI (p = 0.02) compared to healthy individuals (Table 5; Fig. 4).

**Table 3:** Anatomical location of GSD and snare wounds in free-ranging Nubian giraffe *Giraffa camelopardalis camelopardalis* sample from Murchison Falls National Park, Uganda.

Anatomical location	Number of giraffe
GSD	
Neck	16
Neck and chest	1
Neck and forelimb	1
Neck and shoulder	2
Shoulder	1
Snare wound	
Left hindlimb	8
Right hindlimb	1
Right forelimb	4

**Table 4:** Effects of GSD and snare injuries on spatiotemporal limb kinematics in free-ranging Nubian giraffe *Giraffa camelopardalis camelopardalis* from Murchison Falls National Park, Uganda.

Parameter	GSD			Snare		
	F-value	df	P-value	F-value	df	P-value
Relative stride length	1.04	1, 29.1	0.32	11.58	1, 28.8	0.002
Mean stride duration	0.33	1, 22.6	0.57	0.17	1, 22.5	0.68
Relative speed	0.38	1, 26.8	0.54	10.77	1, 26.6	0.003
Limb phase	0.39	1, 31.1	0.54	9.81	1, 30.9	0.004
Mean NSL	1.38	1, 23.0	0.26	0.50	1, 22.8	0.49
Ipsilateral DFAI	0.12	1, 18.5	0.73	8.20	1, 18.3	0.01

**Table 5:** Pairwise comparisons of estimated marginal means for spatiotemporal limb kinematics among different giraffe conditions in free-ranging Nubian giraffe (*Giraffa camelopardalis camelopardalis*). Parentheses contain 95% confidence intervals. Shared superscripts within a row indicate that estimated marginal means were not significantly different.

Parameter	Healthy	GSD	Snare	GSD and snare
Relative stride length	1.24^a^ (1.16 to 1.33)	1.30^a,b^ (1.21 to 1.38)	1.06^c^ (0.96 to 1.17)	1.12^a,c^ (1.03 to 1.21)
Mean stride duration (s)	2.28^a^ (2.16 to 2.39)	2.32^a^ (2.20 to 2.43)	2.31^a^ (2.16 to 2.46)	2.35^a^ (2.23 to 2.47)
Relative speed	0.55^a^ (0.51 to 0.59)	0.56^a^ (0.52 to 0.61)	0.47^b^ (0.41 to 0.52)	0.48^a,b^ (0.44 to 0.53)
Limb phase (%)	12.98^a^ (12.32 to 13.64)	12.73^a,b^ (12.06 to 13.39)	11.71^b,c^ (10.88 to 12.55)	11.46^c^ (10.76 to 12.15)
Mean NSL	2.72^a^ (2.69 to 2.76)	2.75^a^ (2.71 to 2.79)	2.71^a^ (2.66 to 2.76)	2.74^a^ (2.70 to 2.77)
Ipsilateral DFAI (%)	− 0.64^a^ (− 2.78 to 1.50)	− 1.08^a,b^ (− 3.23 to 1.06)	− 4.37^b,c^ (− 7.06 to − 1.68)	− 4.82^c^ (− 7.03 to − 2.60)

### Effect of giraffe condition on neck angle kinematics

**Figure 5:**
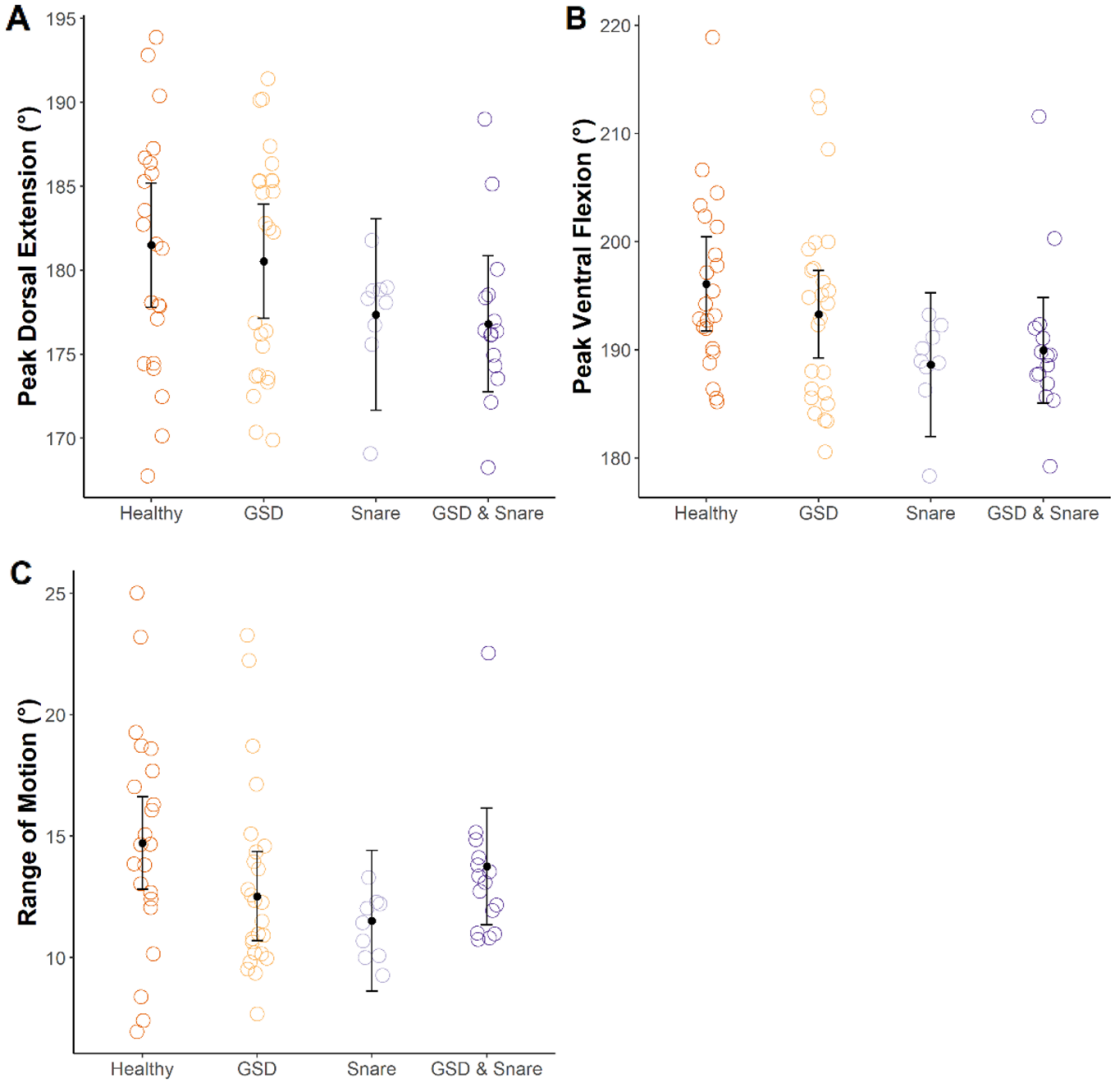
Variation in neck angle kinematics among different conditions (i.e., healthy, GSD, snare wound, and both GSD and snare wound) in free-ranging Nubian giraffe *Giraffa camelopardalis camelopardalis* from Murchison Falls National Park, Uganda. Each point represents a stride. Black dots indicate estimated marginal means and black error bars indicate 95% confidence intervals from the linear mixed models.

The presence of GSD had a significant effect on peak ventral flexion (Table 6); however, posthoc tests revealed no significant differences among groups after controlling for multiple pairwise comparisons (Table 7; Fig. 5).

**Table 6:** Effects of GSD and snare injuries on neck angle limb kinematics in free-ranging Nubian giraffe *Giraffa camelopardalis camelopardalis* from Murchison Falls National Park, Uganda.

Parameter	GSD			Snare		
	F-value	df	P-value	F-value	df	P-value
Peak dorsal extension	0.18	1, 24.1	0.68	3.91	1, 25.9	0.06
Peak ventral flexion	0.39	1, 22.6	0.54	4.46	1, 24.6	0.04
ROM	0.48	1, 20.7	0.50	0.44	1, 29.8	0.51

**Table 7:** Pairwise comparisons of estimated marginal means for neck angle kinematics among different giraffe conditions in free-ranging Nubian giraffe (*Giraffa camelopardalis camelopardalis*). Parentheses contain 95% confidence intervals. Shared superscripts within a row indicate that estimated marginal means were not significantly different.

Parameter	Healthy	GSD	Snare	GSD and snare
Peak dorsal extension (°)	181.4^a^ (178.1–184.7)	180.6^a^ (177.5–183.6)	177.5^a^ (173.4–181.7)	176.7^a^ (173.2–180.2)
Peak ventral flexion (°)	195.3^a^ (191.4–199.2)	193.9^a^ (190.3–197.6)	190.4^a^ (185.4–195.4)	189.0^a^ (184.7–193.2)
ROM (°)	14.0^a^ (12.1–15.9)	13.2^a^ (11.4–15.0)	13.1^a^ (10.6–15.7)	12.4^a^ (10.1–14.6)

## Discussion

We undertook the first in-depth investigation of the spatiotemporal limb kinematics and neck angle kinematics of walking gaits in free-ranging Nubian giraffe. We found that the spatiotemporal limb kinematics of healthy free-ranging giraffe from our sample were generally comparable to those of healthy zoo-housed Masai giraffe both from our preliminary analyses at Cleveland Metroparks Zoo and those reported in Basu et al.^39^. In general, all giraffe used LSLC walking gaits. The average gait cycle of the healthy Nubian giraffe recorded can be described as 66:13 in Hildebrand^40^ terms (i.e., mean duty factor %: limb phase %), which is similar to the 64:15 average gait recorded in our Cleveland Metroparks Zoo sample and the 70:14 average gait recorded by Basu et al.^39^. This suggests that basic kinematic parameters of giraffe walking gaits are conservative across environments and among healthy individuals of different giraffe taxa.

Contrary to our predictions from the knowledge that some Masai giraffe with GSD were found to walk with greater difficulty^18^, the spatiotemporal limb kinematics of Nubian giraffe with only GSD did not differ from those of healthy giraffe. This result is potentially attributable to the fact that GSD lesions were predominantly found on the necks in our sample. GSD primarily affects the necks of the MFNP’s Nubian giraffe population^21^, while GSD lesions are more commonly found on the limbs of giraffe taxa at other sites surveyed to date: e.g., Tanzania (Masai giraffe—Manyara Ranch Conservancy, Selous Game Reserve, Ruaha NP, Serengeti NP, and Tarangire NP) and Namibia (Angolan giraffe—Etosha NP and Puros Conservancy)^17^. It is not yet clear why the population in MFNP is affected primarily on the neck as opposed to the limbs, although ongoing studies by Giraffe Conservation Foundation and partners are being undertaken to evaluate etiology and vector dynamics.

We found that giraffe with snare wounds had shorter stride lengths, slower walking speeds, reduced limb phase values, and more negative ipsilateral DFAI compared to healthy giraffe. Similarly, giraffe with both GSD and snare wounds had reduced limb phase values, and more negative ipsilateral DFAI compared to healthy giraffe. In their study of zoo-housed giraffe, Basu et al.^39^ found that stride frequency (i.e., inverse of stride duration) was consistent across walking speeds and that giraffe increased walking speed by taking longer strides. Our results support this pattern, as giraffe with snare wounds had shorter stride lengths (but consistent stride durations) resulting in slower relative speeds. These kinematic compensations are common in animals with limb or hoof pathologies, and increased ipsilateral asymmetry, in particular, is consistent with a strategy to reduce loading on the injured limb^41,54^. The more negative ipsilateral DFAI values for giraffe with snare wounds indicate shorter contact durations, and potentially reduced peak ground reaction forces and/or impulse, on the side of the injured limb. Recording the ground reaction forces of different limbs throughout stride sequences via force plates could be used to test this assumption—a technique commonly employed to detect lameness in domestic and laboratory animals^60–62^.

The decreased mobility and locomotor efficiency of individuals with snare wounds could have important ramifications for giraffe health and fitness. Giraffe in MFNP travel ~ 14 km per day (M. Brown, pers. comm., May 2021), whilst some individuals, especially subadult and adult males, embark on seasonal migrations related to changes in food availability in the park. As part of these seasonal migrations, individuals may travel up to 30 km between acacia savanna in the wet seasons and broadleaf savanna in the dry seasons^63^. Reduced mobility in affected giraffe may reduce foraging efficiency, limit the extent of seasonal movements, and/or reduce the amount of time giraffe can spend engaging in other behaviors (e.g., resting, breeding, etc.) due to increased time spent locomoting. Impaired mobility may also have negative consequences on important social behaviors. For example, adult males compete for access to females with larger bulls typically out-competing other males^64,65^. Subordinate males may travel in search of other locations where female density is too high for bulls to effectively monopolize^63^. Locomotor deficiencies related to snare wounds and/or GSD may limit dominant males’ ability to mate guard and limit more subordinate males’ ability to travel in search of other breeding opportunities. Finally, although giraffe predation is rare in MFNP^21^, reduced mobility and potential flight ability may make affected individuals more vulnerable to predators. Muneza et al.^27^ examined relationships between GSD and lion predation in Ruaha NP—a site where giraffe are commonly preyed upon by lions. The study documented a positive relationship between severe GSD lesions and signs of attempted lion predation (i.e., bite marks, claw marks, and amputated tails). This suggests lions may preferentially target individuals with severe GSD; however, the authors found no evidence that GSD lesions impacted the likelihood of surviving a lion attack and were not able to record GSD presence or severity on giraffe killed by lions.

Walking gaits were characterized by two oscillations of the neck (one for the left limbs and one for the right limbs). Peak dorsal extension of the neck was consistent with early stance phase of each forelimb and peak ventral flexion occurred at roughly midstance of each forelimb as other have described^35,39^. Basu et al.^39^ modelled neck accelerations and mean ground reaction forces to assess the relationship between the accelerations of the trunk and neck. They found that the horizontal acceleration of neck was largely out of phase (i.e., phase relationship of 23%) with the horizontal acceleration of the trunk, essentially decoupling neck movement from the rest of the body which likely results in significant energy savings. We did not find any significant differences in peak dorsal extension or peak ventral flexion of the neck when comparing animals with GSD and/or snare wounds to healthy individuals. Controlling for speed, neck ROM did not differ among the different condition categories. This is noteworthy given the prevalence of GSD lesions on the neck of giraffe in our sample and suggests that normal neck ROM during walking gaits is attainable despite GSD lesions on the neck.

Future research examining the spatiotemporal gait kinematics of the various giraffe taxa at locations where GSD is common on the limbs is required to better determine the extent to which GSD may affect locomotion. Muneza et al.^22^ found that GSD was especially common on the forelimbs (including cases of unilateral and bilateral lesions) but less common on the hindlimbs of Masai giraffe in Ruaha and Serengeti NPs. This provides an opportunity to examine how forelimb vs. hindlimb, and unilateral vs. bilateral limb lesions impact gait kinematics. Researchers have previously categorized the severity of GSD by estimating lesion sizes^18^ or quantifying lesion sizes via photogrammetry techniques^22^, rather than report presence vs. absence^19,44^ as we have done here due to limitations in our sample size. Another recommended line of research would be to test whether severity of GSD (e.g., mild vs. severe forms) differentially impacts giraffe locomotion.

Overall, we found that MFNP’s Nubian giraffe spatiotemporal limb kinematics and neck angle kinematics were largely conservative between healthy individuals and those with GSD. Our results are consistent with the idea that GSD does not appear to increase mortality of affected giraffe and does not warrant veterinary intervention^44^; however, future study is required to examine locomotor kinematics of giraffe at sites where lesions are found predominantly on the limbs to better assess the potential significance of GSD on giraffe locomotion and associated morbidity and mortality. We found that individuals with snare wounds showed more discernible kinematic compensations consistent with reduced speed and minimized contact on the injured limb. It is likely that some severe snare wounds impact locomotor efficiency and flight capability to the extent that they increase mortality in affected individuals. Furthermore, many animals, including giraffe, do not escape from snare entrapments^30^. In long-term surveys of MFNP, Mudumba et al.^30^ observed the highest density of wire snares recorded to date in sub-Saharan Africa and recorded the remains of fifteen giraffe caught in wire snares. Targeted veterinary de-snaring efforts in MFNP from February 2019 to December 2021 effectively removed snares from 257 live giraffe, emphasizing the severity and scope of this threat (S. Ferguson, pers. comm., April 2021). Ongoing studies in MFNP seek to evaluate the potential fitness costs and impacts of GSD and snare wounds on survival. Locomotor kinematic studies like ours can provide crucial mechanistic perspectives on emergent patterns of larger scale movements and demographic consequences. MFNP supports the largest known population of the critically endangered Nubian giraffe remaining in the world, so identifying and quantifying potential threats to this population is of global consequence for their conservation^42^.

## Supplementary Information

Supplementary Information.

## Data Availability

All data generated or analyzed during this study are included in this published article as a Supplementary Information file.
